# Promoter replacement of *ANT1* induces anthocyanin accumulation and triggers the shade avoidance response through developmental, physiological and metabolic reprogramming in tomato

**DOI:** 10.1093/hr/uhac254

**Published:** 2022-11-15

**Authors:** João Victor Abreu Cerqueira, Feng Zhu, Karoline Mendes, Adriano Nunes-Nesi, Samuel Cordeiro Vitor Martins, Vagner Benedito, Alisdair R Fernie, Agustin Zsögön

**Affiliations:** 1 Departamento de Biologia Vegetal, Universidade Federal de Viçosa, Viçosa 36570-900 MG, Brazil; 2 National R&D Center for Citrus Preservation, Key Laboratory of Horticultural Plant Biology, Ministry of Education, Huazhong Agricultural University, 430070 Wuhan, China; 3 Max-Planck-Institute for Molecular Plant Physiology, 14476 Potsdam, Germany; 4 Division of Plant & Soil Sciences, West Virginia University, Morgantown, WV 26506, USA

## Abstract

The accumulation of anthocyanins is a well-known response to abiotic stresses in many plant species. However, the effects of anthocyanin accumulation on light absorbance and photosynthesis are unknown . Here, we addressed this question using a promoter replacement line of tomato constitutively expressing a MYB transcription factor (*ANTHOCYANIN1, ANT1*) that leads to anthocyanin accumulation. *ANT1*-overexpressing plants displayed traits associated with shade avoidance response: thinner leaves, lower seed germination rate, suppressed side branching, increased chlorophyll concentration, and lower photosynthesis rates than the wild type. Anthocyanin-rich leaves exhibited higher absorbance of light in the blue and red ends of the spectrum, while higher anthocyanin content in leaves provided photoprotection to high irradiance. Analyses of gene expression and primary metabolites content showed that anthocyanin accumulation produces a reconfiguration of transcriptional and metabolic networks that is consistent with, but not identical to those described for the shade avoidance response*.* Our results provide novel insights about how anthocyanins accumulation affects the trade-off between photoprotection and growth.

## Introduction

Anthocyanins are flavonoid pigments that mediate plant-environment interactions and protect plants from abiotic stresses. For instance, in evergreen plants of high latitudes, the photosynthetic machinery is susceptible to photoinhibition due to high irradiance and low temperatures during winter [1]. Under such conditions, anthocyanin accumulation may provide photoprotection [2, 3]. Anthocyanins appeared early in the evolution of land plants and have since diversified biochemically and functionally [4]. They fulfill ecophysiological roles as attractors of pollinators and as antioxidants to protect plants from extreme temperatures, water deficit or high irradiance [5]. However, it is still unclear if their main involvement in the protection of plants facing excessive light is directly as light attenuators or indirectly as antioxidants.

Plants require light as a substrate for photosynthesis, but excessive irradiance can induce damage to the photosynthetic machinery in the thylakoid membranes of the chloroplasts, particularly to photosystem II (PSII) [6]. The damage to PSII and other parts of the photosynthetic apparatus is caused by the excess of reducing power in the electron transport chain, which leads to spurious reduction of oxygen and the formation of reactive oxygen species (ROS) [7]. As anthocyanins have the dual ability of absorbing visible light (500–550 nm) [8] as well as scavenging ROS [9], it is unclear which of these two effects is preeminent in plant photoprotection. A deeper understanding of this question will be beneficial for the biotechnological exploitation of anthocyanins in plant breeding.

One of the difficulties in addressing anthocyanin function is the lack of a model to compare isogenic plants with either green or cyanic leaves. Previous studies have compared either different varieties of the same species or leaves of a single genotype of different age or grown in different conditions [3, 8, 10]. An alternative has been the manipulation of anthocyanin biosynthesis via transgenesis, using heterologous transformation or inducible expression systems [11]. Anthocyanins have industrial application as natural dyes and are also considered as “functional foods” contributing to human nutrition, so the anthocyanin biosynthesis pathway has attracted intense research attention and is relatively well understood, providing suitable targets for genetic engineering [12]. A complex of transcription factors from three families, R2R3-MYB, basic helix–loop–helix (bHLH) and WD40-repeat (WDR) coordinates anthocyanin biosynthesis in most angiosperms studied to date [13]. One of the earliest components identified was *ANTHOCYANIN1* (*ANT1*), a MYB transcription factor of tomato (*Solanum lycopersicum*) [14]. Tomato is an important horticultural crop, and also a genetic model species with a rich repertoire of mutants available for functional studies.

Here, we determined how anthocyanin content affects plant growth and yield and the potential underlying genetic and metabolic implications. *ANT1* expression is associated with upregulation of both early (chalcone synthase, chalcone isomerase) and late (dihydroflavonol reductase; 3-*O*-glycosyltransferase; glutathione S-transferase) anthocyanin biosynthesis enzymes. Transgenic tomatoes with *ANT1* expression driven by the cassava vein mosaic virus (CVMV) promoter were described previously [14]. However, they displayed heterogeneous phenotypes, low expressivity and frequent spontaneous reversion. Instead, we used a tomato line with a TALENs-based targeted insertion of the constitutive 35S promoter upstream of the *ANT1* coding sequence [15]. This genotype provides a consistent, reproducible and stable phenotype of high anthocyanin content in all vegetative organs.

We further used two anthocyanin deficient mutants of tomato cv. Micro-Tom (MT): *anthocyaninless* (*a*) and *anthocyanin absent* (*aa*) [16] to measure a series of developmental, physiological and biochemical parameters. To exclude potential interference from the MT background, which harbours *dwarf*, a brassinosteroid biosynthesis mutation [17], we generated hybrid cyanic plants by crossing MT and *ANT1* to the model tomato cultivar M82. Our results suggest that even in plants grown under high irradiance, anthocyanins alter the light absorption profile of leaves, leading to the shade avoidance response (SAR), a temporary strategy of growth reprogramming to survive under low irradiance conditions [18]. In *ANT1* plants, this suite of traits resulted in impaired growth and reduced yield. We also demonstrate that the gene expression and metabolic signature are consistently altered in cyanic plants compared to green plants. We discuss the implications of these results for the exploitation of increased anthocyanin levels as a crop breeding tool.

## Results

### Anthocyanin overproduction alters plant phenology and development

**Figure 1:**
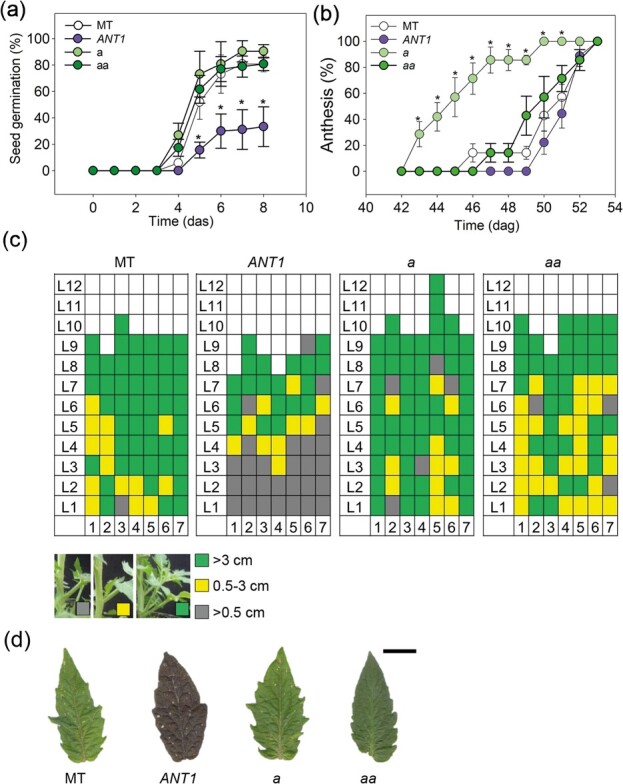
Phenological and developmental alterations caused by anthocyanin accumulation. (a) Seed germination and (b) flowering rates in tomato cultivar Micro-Tom (MT); anthocyanin overproducing *ANTHOCYANIN* (*ANT1*), *anthocyaninless* (*a*) and *anthocyanin absent* (*aa*). (c) Schematic representation of side branching: rows represent leaf position (L1-L12) and columns (1–7) plant replicates. Colors represent the size of the bud as shown in the key. (d) Representative leaflets of each genotype. Scale bar = 1 cm.

We first verified how vegetative growth and phenology are affected by anthocyanin accumulation. The seed germination rate of *ANT1* seeds was reduced compared to that of MT, whereas *a* and *aa* showed a similar germination rate to MT (Figure 1a). After one week, more than 80% of MT, *a* and *aa* seeds had germinated, compared to less than 40% of *ANT1*. The time to flowering was also delayed in *ANT1*, while it is accelerated in *a* and *aa* compared to MT (Figure 1b). Vertical growth was reduced in *ANT1* after flowering but not during the vegetative phase (Table S1, S2). Another important trait that was affected by the anthocyanin content was side branching. We quantified axillary bud development and found that cyanic plants developed less side branches than the other genotypes (Figure 1c). Moreover, total leaf area is severely reduced in *ANT1* compared with the other three genotypes (Figure S1). Lastly, as *ANT1* leaflet margins appear to less serrated (Figure 1d), we quantified terminal leaflet shape using a previously described algorithm [19]. *ANT1* leaflets showed increased circularity, which is inversely proportional to margin indentation (Figure S2 and Table S3).

### Anthocyanin accumulation in tomato reduces fruit yield but not total soluble solids

**Figure 2:**
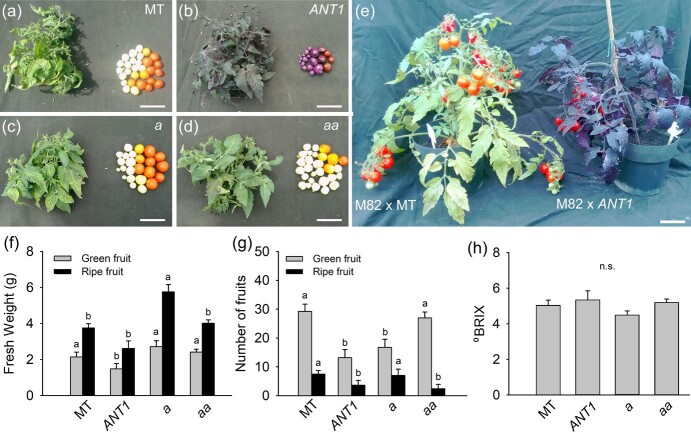
Anthocyanin overproduction reduces tomato yield. Representative plants and fruits of (a) tomato cv. Micro-Tom (MT); (b) anthocyanin overproducing *ANTHOCYANIN* (*ANT1*); (c) *anthocyaninless* (*a*) and (d) *anthocyanin absent* (*aa*). (e) Representative plants of tomato cv. M82 × MT and M82 × MT-*ANT1* hybrids. (f) fresh weight, (g) number of fruits, and (h) °Brix (a measure of the total soluble solids of tomato fruit). Bars show mean ± s.e.m. (n = 6). Significant differences determined by ANOVA followed by Tukey’s test at *p* < 0.05 are shown by different letters.

 We next assessed how anthocyanin accumulation would impact the productivity of *ANT1* plants, both in the MT background (Figure 2a-d) and F_1_ hybrids between cv. M82 × *ANT1* compared to the control M82 × MT (Figure 2e). We verified that anthocyanin overproduction led to consistent phenotypes in the hybrids, such as reduced side branching (Figure S3) and delayed anthesis (Figure S4). In both genetic backgrounds, the anthocyanin accumulators showed a general vegetative growth penalty, with reduced dry weight accumulation, particularly in leaves (Table S4). Fruit yield was similarly reduced in both MT and M82 × MT hybrids (Figure 2f-h and S5). The yield penalty was caused by a simultaneous reduction in both individual fruit size and total number of fruits per plant. On the other hand, total soluble solids were not affected in either genetic background (Figure 2h, Figure S5).

**Table 1:** Photosynthetic parameters derived from A/Cc curves using a biochemical model of photosynthesis [20] for tomato cv. Micro-Tom (MT); ANTHOCYANIN1 overexpressing line (ANT1); anthocyaninless (a) and anthocyanin absent (aa). Values are the means ± s.e.m. (n = 5). The same letter following means indicated no statistical difference based on ANOVA followed by Tukey’s test (P < 0.05)

Genotype/Parameter	MT	*ANT1*	*a*	*aa*
*A_N_* (μmol CO_2_ m^−2^ s^−1^)	22.00 ± 1.57 A	10.93 ± 1.11 B	22.11 ± 0.58 A	21.42 ± 1.10 A
*g_s_* (mol H_2_O m^−2^ s^−1^)	0.40 ± 0.03 A	0.20 ± 0.04 B	0.36 ± 0.05 A	0.41 ± 0.03 A
*g_m_* (mol CO_2_ m^−2^ s^−1^)	0.14 ± 0.01 A	0.05 ± 0.01 B	0.15 ± 0.01 A	0.12 ± 0.01 A
*E* (mmol H_2_O m^−2^ s^−1^)	4.27 ± 0.42 A	2.63 ± 0.34 B	3.93 ± 0.86 A	4.43 ± 0.31 A
*TE (A/g_s_)* (mmol CO_2_ mol^−1^ H_2_O)	55.61 ± 2.81 A	61.64 ± 4.12 A	58.55 ± 3.54 A	53.53 ± 2.88 A
*V*_cmax_ (μmol CO_2_ m^−2^ s^−1^)	95.17 ± 4.50 A	49.54 ± 6.79 B	102.93 ± 9.68 A	82 ± 5.73 A
*J*_max_ (mmol e^−^ CO_2_ m^−2^ s^−1^)	151.03 ± 1.76 A	101.03 ± 2.92 B	152.92 ± 8.23 A	136 ± 1.76 A
*J*_max_ / *V*_cmax_	1.59 ± 0.21 A	2.04 ± 0.31 A	1.5 ± 0.16 A	1.66 ± 0.19 A
*R*_D_ *(*μmol CO_2_ m^−2^ s^−1^)	2.17 ± 0.09 A	1.68 ± 0.05 B	1.87 ± 0.05 A	1.95 ± 0.10 A

^*^ *A*_N_: Net CO_2_ assimilation rate at 400 ppm CO_2_ and 1000 μmol.m^−2^.s^−1^; *g*_s_: stomatal conductance; *g*_m_: mesophylic conductance; *E:* transpiration,: WUE-Water Use Efficiency; *V*_cmax_: maximum carboxylation capacity; *J*_max_: maximum capacity for electron transport rate.

**Table 2:** Photosynthetic parameters from light-response curves in tomato cv. Micro-Tom (MT); ANTHOCYANIN1 overexpressing line (ANT1); anthocyaninless (a) and anthocyanin absent (aa) determined in ambient CO2 (400 ppm) at 25°C. Values are the means ± s.e.m. (n = 5). The same letter following means indicated no statistical difference based on ANOVA followed by Tukey’s test (P < 0.05)

Genotype/ Parameter	MT	*ANT1*	*a*	*aa*
*A_PPFD_* (μmol CO_2_ m^−2^.s^−1^)	16.7 ± 0.39 A	8.8 ± 0.82 B	19.5 ± 0.97 A	18.4 ± 1.31 A
*I*_c_ (μmol CO_2_ m^−2^.s^−1^)	16.4 ± 0.64 C	24.7 ± 2.40 B	21.4 ± 0.70 BC	32.4 ± 2.86 A
*I*_s_ (μmol CO_2_ m^−2^.s^−1^)	460.5 ± 30.23 A	557.6 ± 22.08 A	663.7 ± 70.80 A	577.7 ± 58.70 A
1/Φ (μmol mol^−1^ CO_2_)	20.1 ± 0.86 A	21.6 ± 2.41 A	15.6 ± 0.42 AB	11.6 ± 1.51 B

a *A*_PPFD_: Net CO_2_ assimilation rate saturated by light; *I*c: light compensation point; *I*s: light saturation point; 1/Φ:1/ Light use efficiency.

### Anthocyanins alter light absorbance and leaf structure

**Figure 3:**
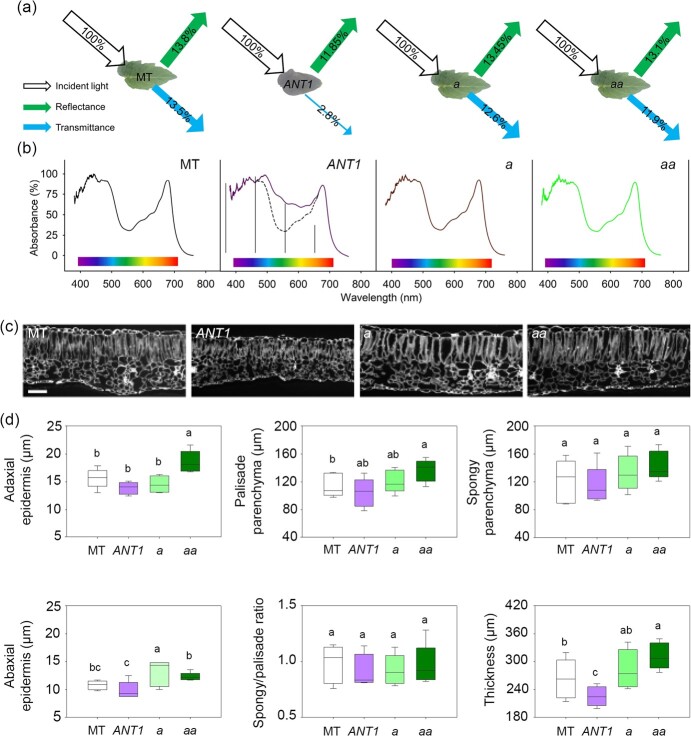
Constitutive anthocyanin production alters light absorbance. (a) Summary diagram showing leaf spectral properties. Percentage incident light (white arrow), reflectance (green arrow) and transmittance (blue arrow) in tomato cv. Micro-Tom (MT); anthocyanin overproducing *ANTHOCYANIN1* (*ANT1*); *anthocyaninless (a)* and *anthocyanin absent* (*aa*). (b) Percentage absorbance of light as a function of incident wavelength on the same genotypes. The *ANT1* curve shows an overlay of the MT curve as a dotted line and the spectra of blue (400–500 nm), green (500–600 nm) and red (600–700 nm) delimited by vertical lines in the ANT1 curve. The dotted curve represents the values for MT for comparison. Curves are means of n = 5 plants. (c) Representative cross-sections of leaves. Scale bar = 100 μm. (d) Box-plots showing measurements of adaxial epidermis, palisade parenchyma, abaxial epidermis, spongy/palisade parenchyma ratio, and whole lamina thickness. Measurements obtained from the fourth fully expanded leaves at 60 DAS (n = 5). Significant differences by ANOVA followed by Tukey’s test at *p* < 0.05 are shown by different letters.

Given that plant phenology and yield are associated with leaf light absorbance and photosynthesis, we analyzed the effect of anthocyanin accumulation on light absorbance and leaf structure. The leaves of *ANT1* plants had more than 12-fold the amount of anthocyanins found in MT (Figure S6). In the anthocyanin-deficient mutants *a* and *aa*, as expected, anthocyanin content was not detectable (Figure S6). Given these alterations, we first determined the spectral properties of leaves in the genotypes. When exposed to white light, all green leaves had a similar proportion (around 13%) of reflectance and transmittance (Figure 3a). The deep purple leaves of *ANT1* plants, on the other hand, showed a drastic decrease in transmittance (2.8%), with only a minimal decrease in reflectance. As this result implied increased absorbance, we conducted a more detailed analysis of this parameter through the entire spectrum of visible light (300–700 nm). As expected, all leaves showed peaks of absorbance in the blue (around 450 nm) and red (around 650 nm) spectra, matching those of the chlorophyll molecules (Figure 3b). However, compared to the green leaves of MT, *a* and *aa*, the purple leaves of *ANT1* did not show a deep valley in absorbance between 500–600 nm in the yellow-green region, which is in line with the peak absorbance of anthocyanins (around 500 nm). However, the higher absorbance of *ANT1* leaves also included the long-wave end of the blue spectrum (475–500 nm) and the short-wave end of the red (600–650 nm). We hypothesized that this increased absorbance could lead to heating of the leaf, so we determined leaf temperature over the course of a day in a glasshouse. Indeed, there was an increase in energy dissipation in the form of heat in cyanic plants at the hottest hours of the day, between 12:00 and 16:00 (Figure S7).

We next focused our attention on leaf structure, by analyzing leaf cross-section micrographs (Figure 3c). *ANT1* leaves were thinner than MT ones, whereas *a* and *aa* leaves were thicker (Figure 3c). To isolate the contribution of each tissue to this difference, we measured the thickness (as vertical length) of the adaxial and abaxial epidermes and palisade and spongy parenchymas (Figure 3d). We found that the differential elongation of the palisade parenchyma (Figure 3d) and reduced thickness of both epidermes were responsible for the leaf thickness changes among the genotypes. Both the altered light absorbance profile and leaf structure suggested that photosynthesis could be impaired in cyanic leaves, so we next performed a series of gas exchange analyses on the genotypes.

### Plants with higher anthocyanin content have altered gas exchange properties

**Figure 4:**
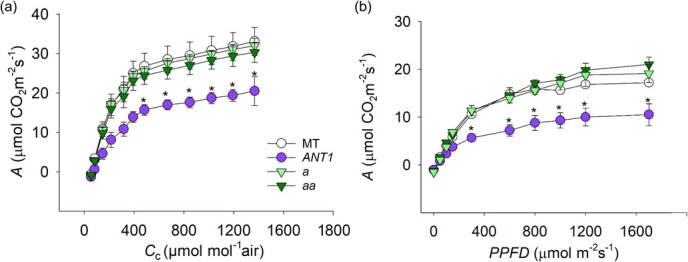
Photosynthesis properties are altered by anthocyanin accumulation. Tomato cv. Micro-Tom (MT); anthocyanin overproducing *ANTHOCYANIN* (*ANT1*)*; anthocyaninless* (*a*) and *anthocyanin absent* (*aa*). Response curves of CO_2_ assimilation (*A*) to changes in (a) ambient CO_2_ concentration (curve measured at 1000 μmol photon m^−2^ s^−1^), and (b) *PPFD* at 400 ppm of CO_2._ Each point represents mean ± s.e.m. (n = 4) and ^*^ indicates significant differences by Tukey’s test at *p* < 0.05.

To determine whether anthocyanin accumulation caused photosynthetic limitations, we examined the response of the *A_N_* to chloroplast partial pressure of CO_2_ (*C*_c_) under saturating light (*A_N_ / C*_c_ curves) and to photosynthetically active photon flux density (*PPFD*) under ambient CO_2_ (*A_N_ / PPFD* curves, Figure 4a-b). Using the data from the curves and a biochemical model for photosynthesis [20], we estimated maximum Rubisco carboxylation rate (*V*_cmax_) and electron transport rate (*J*_max_) (Tables 1 and 2). At ambient CO_2_ (400 ppm), *A_N_* was reduced by half in *ANT1* plants compared to MT (10.93 ± 1.11 v*.* 22.00 ± 1.57 μmol CO_2_ m^−2^ s^−1^, *p* = 0.004), as was *g*_s_ (0.20 ± 0.04 v. 0.40 ± 1.11 mol H_2_O m^−2^ s^−1^, *p* = 0.0001). The anthocyanin deficient mutants did not show any differences with MT in either *A_N_* or *g*_s_ (Table 1). Both *V*_cmax_ and *J*_max_ were depressed in *ANT1* plants but not in *a* or *aa*. The dark respiration rate (*R*_D_) was lower in *ANT1* than in the other three genotypes.

The information from the *A*_N_/*PPFD* curves demonstrated that *ANT1* assimilation of light-saturated CO_2_ was half that of the other genotypes (Table 2). We also calculated the light compensation point (*I*_c_), *i.e.* the light intensity at which the *A*_N_ is zero. The value was highest for the *aa* mutant and lowest for MT, with intermediate values for *ANT1* and *a* (Table 2). All genotypes showed a similar light saturation point (*I*_s_), and the light utilization (*i.e.* the reciprocal of the maximum quantum yield) was highest in MT and *ANT1*, with lower values in *a* and *aa* (Table 2). In summary, all these analyses indicated that anthocyanin accumulation altered the development and physiological status of the plant, leading to shade avoidance response (SAR) (Table 3).

**Table 3:** Canonical shade avoidance response (SAR) traits and the ANT1 phenotype. SAR trait changes in response to shade as described in the literature [18, 19, 21–25] compared to the phenotype of ANTHOCYANIN1 (ANT1) overexpressing plants

**Traits**	**Response to shade**	**Phenotype in *ANT1***
Seed Germination	Delayed	Delayed
Extension growth	Accelerated	Accelerated
Internode extension	Increased	Increased
Etiolation (height:diameter ratio)	Increased	Increased
		
Leaf development	Delayed	Delayed
Number of leaves	Reduced	Reduced
Leaf area growth	Reduced	Reduced
Leaf thickness	Reduced	Reduced
Leaf shape	Altered	Altered
		
Chloroplast development	Delayed	Not determined
Chlorophyll concentration	Increased	Increased
Chlorophyll *a*:*b* ratio	Altered balance	Increased
		
Apical dominance	Increased	Increased
Side branching	Inhibited	Inhibited
		
Flowering	Accelerated	Accelerated
Rate of flowering	Increased	Increased
Seed set	Reduced	Not determined
Fruit development	Reduced	Reduced
		
Carbon assimilation	Reduced	Reduced
Photosynthetic rate	Reduced	Reduced
Biomass	Reduced	Reduced
Vegetative:reproductive ratio	Reduced	Reduced

### Elevated anthocyanin content provides photoprotection in response to high irradiance

**Figure 5:**
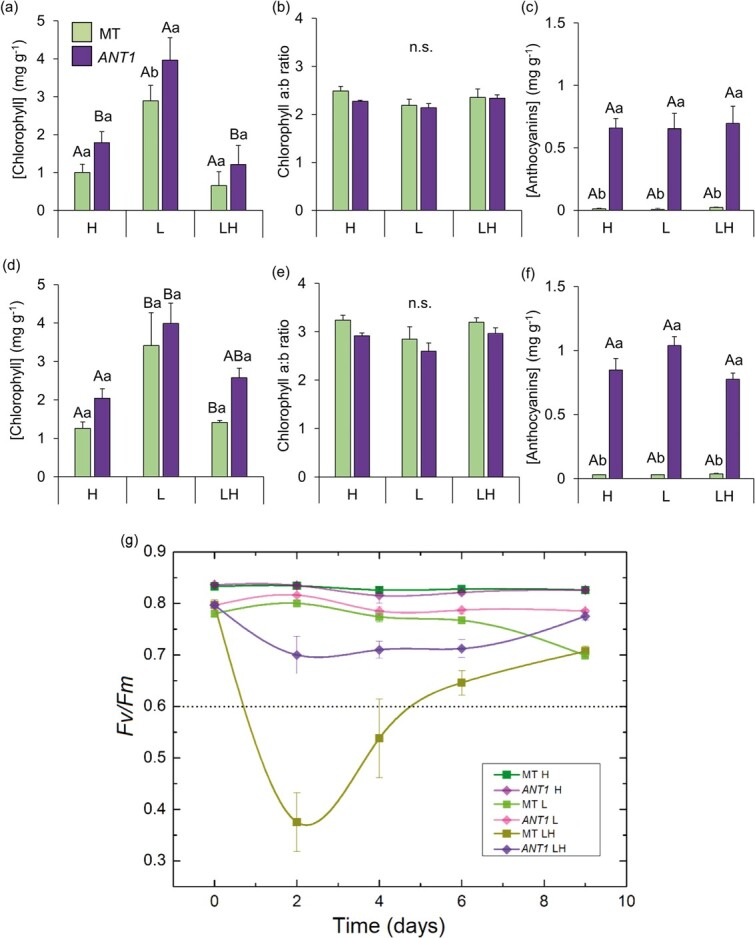
Elevated anthocyanin content provides photoprotection to high irradiance. Pigment contents in tomato cv. Micro-Tom (MT) and the anthocyanin overproducer (*ANT1*) grown under continuous high light (H, 1000 μmol m^−2^ s^−1^), continuous low light (L, 100 μmol m^−2^ s^−1^) or a step-change from L to H treatment (LH). Pigment contents in old, fully developed leaves in the initial light treatment (a-c) or new leaves developed after the irradiance step-change in the LH treatment (d-f). (g) Time course of maximum PSII quantum efficiency (F_v_/F_m_). Bars or dots show mean ± s.e.m. (n = 8). Significant differences by ANOVA followed by Tukey’s test at *p* < 0.05 are shown by different letters (capital letters refer to comparisons between treatments of the same genotype, and lower case letters between genotypes of the same treatment).

As SAR may lead to susceptibility to high light stress, we grew MT and *ANT1* plants first at low irradiance (L, 150 μmol photon m^−2^ s^−1^) and then exposed them to high irradiance (H, 1500 μmol photon m^−2^ s^−1^) at 35 days of age to determine the potential effect of anthocyanins in modulating the response to light stress. Plants remained in this H condition until new leaves were developed and then harvested for pigment determination and compared to older leaves developed in the L condition (Figure 5a-f). Total chlorophyll levels tended to be higher in *ANT1* plants in both “old” and “new” leaves, with no difference of chlorophyll *a*:*b* ratio (Figure 5). As expected, total anthocyanins were consistently higher in *ANT1* than in MT. We also carried out a time-course analysis of *F*_v_/*F*_m_ (Figure 5g). MT leaves showed a severe reduction in *F*_v_/*F*_m_ (from an initial value of 0.8) already at the first measure (two days) after the irradiance swap. Subsequently, *F*_v_/*F*_m_ values increased and stabilized at a lower value (around 0.7). *ANT1*, on the other hand, maintained higher *F*_v_/*F*_m_ values (above 0.7) at all time points after the swap (Figure 5g). These results further confirmed that the anthocyanin accumulation not only induces SAR but also protects photosynthesis against the high irradiance stress.

### Expression patterns of genes related to shade avoidance response are altered in anthocyanin-accumulating plants

**Figure 6:**
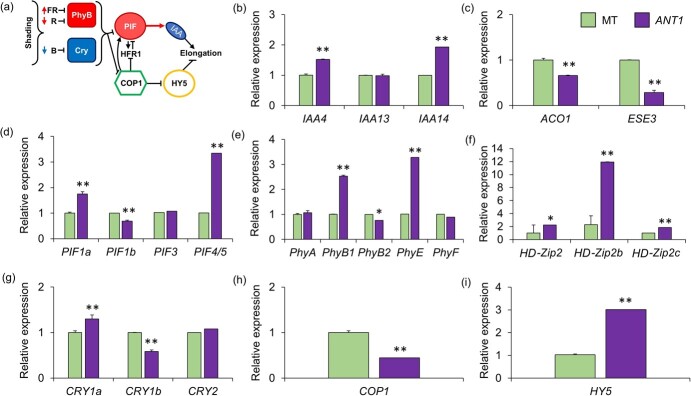
Shade-avoidance response-related gene expression is altered by anthocyanin accumulation. (a) Interaction among the main modulators of light response due to shading. (b-i) The expression levels of genes of the shade avoidance response in tomato cv. Micro-Tom (MT) and the anthocyanin overproducer (*ANT1*). (b) *PHYTOCHROME* (*Phy*) *A*, *B1*, *B2*, *E* and *F*; (c) *CRYPTOCHROME* (*CRY*) *1a*, *1b* and *2*; (d) *CONSTITUTIVE PHOTOMORPHOGENIC 1* (*COP1*), (e) *PHYTOCHROME-INTERACTING FACTORS* (*PIFs*) *1a*, *1b*, *3* and *4/5*; (f) *ELONGATED HYPOCOTYL 5* (*HY5*) (g) *Auxin/Indole-3-Acetic Acid* (*IAA*) *4*, *13* and *14*; (h) *HOMEODOMAIN LEUCINE ZIPPER 2* (*HD-Zip2*), *2b* and *2c*. The analyses were conducted on RNA extracted from fully expanded leaves in biological triplicates using *ACTIN*, *TIP41* and *CAC* as endogenous controls for relative expression normalized to MT. Bars show mean ± s.e.m. (n = 3). Significant differences determined by ANOVA followed by Tukey’s test at *p* < 0.05 (^*^) or *p* < 0.001 (^**^). Model in panel (a) was redrawn from [18].

Since the transcriptomic signature of plants growing in the shade has been well established [18] (Figure 6a), we extracted RNA from fully-expanded leaves of MT and *ANT1* plants and analyzed SAR-related gene expression. Qualitative changes in irradiance are perceived by phytochromes (Phy) for the Red:Far Red (R:FR) wavelengths and cryptochromes (Cry) for the Blue (B) ones. Tomato has five *Phy* and three *Cry* gene paralogues – we found higher *PhyB1*, *PhyE* and *Cry1a* and lower *PhyB2* and *Cry1b* expression in *ANT1* compared to MT plants (Figure 6 e,g). *CONSTITUTIVELY PHOTOMORPHOGENIC 1* (*COP1*) forms part of a complex that stabilizes *PHYTOCHROME INTERACTING FACTORS* (*PIFs*), the main positive molecular triggers of SAR. Both *COP1* and *PIF1b* expression is reduced in *ANT1*, whereas *PIF1a* and *PIF4/5* expression is increased in this genotype (Figure 6d, h). The transcription factor *ELONGATED HYPOCOTYL 5* (*HY5*) is a target of repression by *COP1* and controls responses to light and temperature. *HY5* expression was induced while that of COP1 was reduced in *ANT1* plants compared to MT (Figure 6f,i). Lastly, downstream effectors of SAR include *Auxin/Indole-3-Acetic Acid (IAA)*, of which *IAA4*/*14* was upregulated in *ANT1 along with* three *HD-Zip2* homeobox transcription factors (Figure 6b, f).

### Primary metabolism signatures are affected by anthocyanin accumulation in two different genetic backgrounds

**Figure 7:**
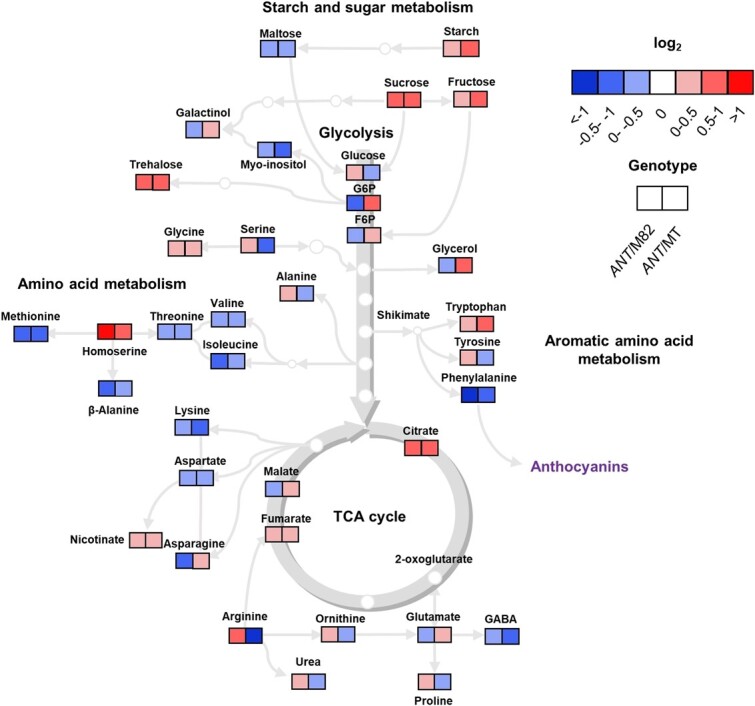
Primary metabolism is reconfigured by anthocyanin overproduction. Heat map of relative primary metabolite concentrations on fully expanded leaves of the anthocyanin overproducer (*ANT1*) relative to either tomato cv. M82 (M82) or Micro-Tom (MT). Each square represents the ratio between *ANT1* and its respective wild-type (n = 6). The color key indicates the relative value (red = higher, blue = lower).

We also analyzed relative primary metabolite levels in leaves of *ANT1* (in MT background) and the M82 × *ANT1* hybrids. A consistent pattern was observed for some carbohydrates: sucrose, fructose and trehalose concentrations were increased in *ANT1* plants, whereas maltose and myo-inositol were lower (Figure 7). With the remarkable exceptions of homoserine and tryptophan, both upregulated in *ANT1*, most amino acid levels were decreased in cyanic leaves, including phenylalanine, branched-chain amino acids (valine, isoleucine), threonine, lysine, methionine and aspartate. Among the organic acids, both citrate and fumarate were mildly upregulated in *ANT1* plants (Figure 7).

## Discussion

We analyzed an anthocyanin overproducing transgenic tomato line, *ANT1*, previously produced via targeted TALENs-mediated insertion of a constitutive (CaMV35S) promoter upstream of the start codon of the *ANT1* gene in chromosome 10 [15]. Unlike conventional transgenics with random T-DNA insertions, which are prone to gene silencing, transgene suppression and variable expressivity (leading to phenotypic differences between individuals) due to positional effects of the T-DNA in the genome and non-Mendelian inheritance [26], this line shows a stably inherited, consistently reproducible phenotype of increased anthocyanin production.

### Elevated anthocyanins alter light absorption profile in leaves and induce traits of SAR

*ANT1* accumulated anthocyanins, driving a significant decrease in light transmittance and alterations in spectral quality that include higher absorbance in the red, green and blue wavelengths. Light quantity and quality are interpreted by plants as developmental signals for photomorphogenesis. This poses a research challenge to study cyanic plants, as it is hard to disentangle the effects of anthocyanins on photosynthesis and on photomorphogenesis [27]. Shade-intolerant plants, such as tomato, perceive neighbor proximity as a reduction in R:FR ratio, which triggers SAR, a suite of developmental and physiological responses that allow acclimation to shading [18].

*ANT1* plants displayed suppressed side branching, reduced leaf area, rounder leaves and lower biomass accumulation, among other classical SAR traits (summarized in Table 3) even when grown under high irradiance [2]. As expected, *ANT1* plants showed strong absorbance in the green (500–600 nm) region of the visible light spectrum, but also some absorbance in the higher end of the blue (475–500 nm) band, close to the blue absorption peak of chlorophyll *b* and on the lower end of the red (600–650 nm) band [27]. SAR in anthocyanin-rich plants could thus be caused by either the perception of green-enriched light [28] or a lower red-to-far red ratio (R:FR) [29]. All these types of spectral alterations are found in the subcanopy and understory of a forest, or may be caused by shading by taller plants [30]. The potential contribution of blue, green and red light absorption by *ANT1* plants could be resolved with further studies analyzing their responses to altered light quality. We noticed that increased light absorption by anthocyanins raised leaf temperature during the hottest time of the day, which concurs with previous work connecting low ambient temperatures with increased anthocyanin biosynthesis [10, 31].

We also found lower photosynthetic CO_2_ assimilation rates in *ANT1* plants through a strong biochemical limitation to photosynthesis: both maximum Rubisco carboxylation rate (*V*_cmax_) and electron transport rate (*J*_max_) were severely depressed in the cyanic plants. Chlorophyll concentration was not reduced nor was chlorophyll *a*:*b* ratio increased in *ANT1*, as is typical of shaded plants [25]. Chlorophyll content is strongly positively correlated with photosynthetic capacity, so this suggests that the limitation to photosynthesis in *ANT1* is not biochemical but could be related to leaf structure [32]. *ANT1* plants have double the specific leaf area (SLA) of MT, so they have less photosynthetic machinery per unit leaf area [33]. Thinner leaves were also described in a purple cultivar of basil (*Ocimum basilicum*), leading to lower *A* rates [3].

Fruit yield in *ANT1* plants in both backgrounds, MT and the M82 × MT hybrid, was significantly reduced, through a combination of reduced fruit size and number. Further analyses revealed that side branching is strongly suppressed in cyanic plants. Since both MT and M82 are determinate plants harboring the *self-pruning* mutation [17, 34], vertical growth terminates after flowering and continues laterally by activation of axillary buds. A large part of the vegetative and reproductive growth in determinate tomatoes derives from side branches [35], which partly explains the severely reduced total leaf area and fruit yield in *ANT1* plants. However, the content of total soluble solids in the fruit was not changed between genotypes, but the individual fruit size was reduced in *ANT1* plants.

Interestingly, many of the SAR traits, such as the strong inhibition of side branching, increased branch-to-stem diameter ratio and altered leaf shape in *ANT1* plants are all phenomena that could be associated with the phytohormone auxin [36]. Indeed, growing evidence suggests the existence of an intimate link between SAR and auxin signaling [37, 38]. Some reports show that anthocyanins and auxin metabolism are linked such that increased levels of auxin decrease anthocyanin levels [39]. Previous work has also shown that flavonoids play negatively roles in polar auxin transport [40]. Nevertheless, how the constitutive production of anthocyanins influences auxin metabolism remains unknown. More recent work has implicated jasmonate, salycilic acid, cytokinins and ethylene in the reprogramming of tomato morphogenesis under low light [41]. The MT background is an excellent model to explore this question, as a collection of hormone mutants is already available and future crosses of these mutants with *ANT1* plants may help to unravel the relationship of anthocyanins and auxin [42].

### Transcriptional and metabolic signatures of anthocyanin-enriched plants

Tomato harbours five genes (*PhyA*, *PhyB1*, *PhyB2*, *PhyE* and *PhyF*) that encode the apoprotein portion of the respective phytochromes [43]. Phytochromes can absorb both R (~660 nm) and FR (~730 nm) light and function as transcriptional regulators by interacting with the promoter regions of downstream target genes. In response to high R:FR ratios, they suppress the shade response by antagonizing of PHYTOCHROME-INTERACTING FACTORs (PIFs), which otherwise regulate most physiological aspects of SAR. Blue light (B) signaling also converges on PIFs, as the B receptors, cryptochromes (Cry) and phototropins (phot) are also negative regulators of PIFs in response to high B intensity. *ANT1* plants displayed opposite expression levels between *PhyB1* and *PhyB2*, which were respectively higher and lower than MT control plants. The tomato *B1* and *B2* paralogues show both common and divergent roles [44]. Notably among the latter is the R-induced anthocyanin biosynthesis in seedlings, which shows a wild-type pattern in a *phyB2* mutant but is strongly suppressed in the *phyB1* mutant [44]. The most conspicuous difference, however, was the three-fold higher *phyE* expression in *ANT1* compared to MT. PhyE functions redundantly with PhyB1 and PhyB2 in the control of shade avoidance [45].

Shading leads to activation of PhyB, triggering accumulation of the E3 ligase CONSTITUTIVE PHOTOMORPHOGENIC1 (COP1) in the nucleus [46] and an increased stability of the basic leucine zipper (bZIP) ELONGATED HYPOCOTYL5 (HY5) [47]. Our results, however, show reduced expression of COP1 and increased expression of HY5. Interestingly, HY5 plays positive role of anthocyanin biosynthesis [48]. As mentioned above, the main mediator of shade responses is the degradation of PIFs. The most significant difference in PIF expression was found for PIF4/5, which was increased more than three-fold in *ANT1* compared to MT. *PIF4/5* expression is induced by dark treatment in tomato [49] and in Arabidopsis, which genome contains two orthologs, *PIF4* and *PIF5*. PIF4 and PIF5 negatively regulate the anthocyanin biosynthesis in response to red light and trigger ethylene biosynthesis and ethylene-induced senescence in the dark [50]. However, we found that expression of the shade-induced ethylene biosynthesis gene *ACO1* and the ethylene effector *ESE3* to be reduced [37]. Moreover, the expression of two *Aux/IAA* genes and the three tomato orthologs of Arabidopsis *ATHB2* (*ARABIDOPSIS THALIANA HOMEOBOX PROTEIN 2*) was increased, which is usually observed upon shading [3, 19]. Although the transcriptional levels are consistent with *ANT1* plants behaving as shaded plants, many of the molecular players involved in SAR are regulated at the post-transcriptional level, via degradation or post-translational modification.

Lastly, we analyzed the relative levels of primary metabolites between cyanic plants and their respective controls. We found that anthocyanin-accumulating genotypes had increased starch and reduced maltose levels in their leaves, which agrees with the reduced respiration rate observed in *ANT1*. A buildup of sucrose, trehalose and fructose was also observed in cyanic leaves. Anthocyanin biosynthesis is stimulated by a sucrose-specific signaling pathway that converges on the transcriptional upregulation of *MYB75*/*PAP1*, which is also activated by HY5 [51]. High levels of hexoses in source leaves due to reduced translocation to sinks can lead to photosynthetic downregulation via feedback inhibition [52]. This result agrees with the reduced number of leaves and fruits in *ANT1* plants, which translates into tissues with weaker sink strength and lower photosynthate demand [53]. Phenylalanine level was lower in cyanic plants, possibly indicating a more active phenylpropanoid biosynthetic pathway in these genotypes [54]. The early biosynthesis genes such as chalcone synthase and chalcone-flavonone isomerase, and late genes of the flavonoid pathway are all activated by the ANT1 transcription factor [55]. The main bottleneck in tomatoes is the normally suppressed expression of *CHI*, which is located downstream of phenylalanine. However, previous work has indicated mixed evidence on changes in phenylpropanoid metabolism in cyanic and cyanic leaves [2, 56, 57].

## Conclusion

Anthocyanins are highly conspicuous pigments with major effects on plant physiology. We have shown that a cyanic tomato produced by stable integration of a strong promoter via genome engineering, coupled with anthocyanin-deficient mutants, all in the same genetic background, constitute a powerful tool to address some of the outstanding questions in anthocyanin research. Our results show that anthocyanins absorb light in the blue and red bands of the spectrum and develop traits of the shade avoidance response frequently observed in plants growing in the shade. Through targeted transcriptomic and metabolomic analyses we further show that anthocyanin accumulation produces a reconfiguration of both networks that is consistent but not identical to the shade avoidance response. Yield is limited by a simultaneous reduction in source (less and thinner leaves) and sink (less and smaller fruit) organs. Further research using this system may contribute to a better understanding of anthocyanin function and enable their biotechnological manipulation for plant breeding.

## Material and methods

### Plant material

Tomato (*S. lycopersicum*) cv. Micro-Tom (MT), heterozygous for *35S::ANT1^TAL-2^* (anthocyanin overexpressing plants with a TALENs-based insertion of a 35S promoter upstream of the *ANTHOCYANIN1* gene – hereafter *ANT1*), *anthocyaninless* (*a*) and *anthocyanin absent* (*aa*) were used in the present study. Generation of the *ANT1* line was described previously [15]*.* Seeds of *ANT1* were kindly provided by Prof. Dan Voytas (University of Minnesota, USA) and seeds of *a* and *aa* introgressed into the MT background (BC6Fn) were donated by Prof. Lázaro Peres (University of São Paulo, Brazil). Crosses between *ANT1* × cv. M82 and MT × cv. M82 were performed to generate F_1_ plants with either increased or normal anthocyanin content in a tomato cultivar (M82) of large size. Seeds were germinated and cultivated in a greenhouse in Viçosa, Minas Gerais, in southeastern Brazil.

Genotypes in the MT background were grown from May to August 2017 at a temperature of 30°/25°C, repeated in September to December 2017 . A step-change in irradiance intensity experiment was conducted on plants in the MT background cultivated from September to December 2018. Plants were germinated and cultivated in the greenhouse under high light (H) conditions (maximum of 1500 μmol photons m^−2^ s^−1^). For the irradiance swap analysis, they were cultivated in the same conditions but covered with neutral shade cloth to produce low (L) irradiance (maximum of 150 μmol photons m^−2^ s^−1^) with no alteration of spectral quality. At 35 days after germination, the shade cloth was removed (LH treatment). Irradiance data throughout a representative day for both conditions are shown in Figure S8. *F*_v_ and *F*_m_ were measured with the fluorescence probe mini-PAM II, WALZ (Effeltrich, Germany). Another experiment was conducted to assess the effect of anthocyanin accumulation in a M82 × MT hybrid plants grown from September to December 2017 in the same conditions.

### Anatomical analyses

All anatomical analyses were performed on 60 day old plants. The central leaflets of the fifth leaf were sampled and fixed in formaldehyde, acetic acid, alcohol (FAA) solution under a vacuum of −20 in Hg for 48 h at room temperature. The samples were dehydrated in an ethanol series (70, 85 and 95%) for 2 h in each mixture under a vacuum of −20 in Hg and pre-infiltrated with a methacrylate solution (Historesin-Leica) and remained in 95% ethanol (1:1) for three days under vacuum for 2 h a day. The material was included in methacrylate (Historesin-Leica), according to the manufacturer’s information. On a 5 m thick autofocus rotary microtome (model RM2155, Leica Microsystems Inc., Deerfield, USA), the samples were cut into cross-sections and stained with toluidine blue [58]. The substance was imaged using Olympus Optical (model AX-70 TRF) connected to an image-capture computer with Zeiss AxioCam. Software called Image-Pro ® Plus was used to measure the images.

### Leaf optical properties and temperature determinations

The spectra of reflectance (*R*) and transmittance (*T*) in the adaxial face of fully-expanded leaves (n = 5 per genotype) were measured by throughout the 280–880 nm spectrum using a Jaz Modular Optical Sensing Suite portable spectrometer. The absorbance (*A*) spectrum was calculated following the Lambert–Beer law [59]. Leaf temperature was measured with a digital infrared thermometer (B-MAX Industrial model) at a distance of five cm from the leaf surface. The same leaflet used to determine gas exchange was used for all determinations.

### Gas exchange analyses

The net CO_2_ assimilation rate (*A_N_*) and stomatal conductance (*g*_s_) were determined along with chlorophyll *a* fluorescence parameters using a portable open-flow gas exchange system (LI-6400XT, LI-COR, Lincoln, NE, USA) equipped with an integrated fluorescence chamber head (LI-6400-40, LI-COR Inc.). The gas exchange parameters were measured as described [60]. The photosynthetic light response (*A/PPFD*) and internal CO_2_ partial pressure (*A/C_i_*) response curves were obtained as described [61]. Furthermore, taking as a basis the data obtained by the *A/C_i_* curves we were able to estimate mesophyll conductance (*g_m_)* and CO_2_ in chloroplastic stroma (*C_c_*) [62].

### Pigment analyses

Pigments were quantified on the same leaves used for chlorophyll fluorescence determinations. Chlorophyll was extracted in acetone and measured as described previously [63]. Total anthocyanin determination was performed following a standard protocol [64].

### Metabolic profiling

Leaf samples were used for metabolite profiling with the extraction performed using the MTBE method [65]. 200 μL of dried polar phase was resuspended and derivatized in methoxyaminhydrochloride and then incubated in *N*-methyl-*N*-[trimethylsilyl] trifluoroacetamide (MSTFA). The detailed analysis was performed following the recommendations of Salem et al. [65].

### Starch content analysis

After metabolite extraction, the pellet was washed with 80% ethanol. After washing with water and drying with SpeedVac, the pellet was incubated with 0.5 ml of 200 mM sodium acetate at 95°C for 1 h. Digested starch is broken down into glucose monomers by the mixture of α-amyloglucosidase and α-amylase enzymes. The determination of glucose concentrations was performed as described [65].

### RT-qPCR experiment

TRIzol reagent (Invitrogen, Waltham, MA, USA) was use to extracted total RNA from leaves and the first-strand cDNA was synthesized using PrimeScript RT Reagent Kit with gDNA Eraser (Takara). RT-qPCR was carried and analyzed as described [66] using the primers listed in Table S5.

### Statistical analyses

All statistical analyses were carried out using the R software statistical package version 3.5.1 (R Development Core Team, 2011). The data were subjected to analysis of variance ANOVA, subjected to Shapiro–Wilk test (5%) to prove normality and the means were analyzed by the Tukey’s test.

## Acknowledgments

AZ was supported by a CAPES/Alexander von Humboldt Foundation Experienced Researcher Fellowship (88881.472837/2019-01) and Foundation for Research Assistance of the Minas Gerais State (FAPEMIG, Brazil, APQ-01942-22). FZ was supported by the Major Special Projects and Key R&D Projects in Yunnan Province (202102AE090020 and 202102AE090054). We thank Prof Lázaro E. P. Peres (University of São Paulo) and Prof Fábio M. DaMatta (Federal University of Viçosa) for valuable suggestions on early stages of the project.

## Author contributions

JVAC, FZ carried out experiments and analyzed the data. SCVM, ANN and AZ helped to prepare materials and analysis tools. AZ, VB and ARF designed the experiments and wrote the paper with contributions from the other authors.

## Data availability

All data supporting the findings of the present study are available within the paper and its supplementary material files.

## Conflict of interest

No conflict of interest declared.

## Supplementary data

Supplementary data is available at *Horticulture Research* online.

## Supplementary Material

Web_Material_uhac254
