# Supplementary material for: Promoter replacement of ANT1 induces anthocyanin accumulation and triggers the shade avoidance response through developmental, physiological and metabolic reprogramming in tomato
Source: Hortic Res. 2022 Nov 15;10(2):uhac254. doi: 10.1093/hr/uhac254 (PMC9896602; doi:10.1093/hr/uhac254)
Supplement: Web_Material_uhac254 [file web_material_uhac254.zip › Supporting Information_SUBMIT.docx]

**Supporting Information for Cerqueira et al.**


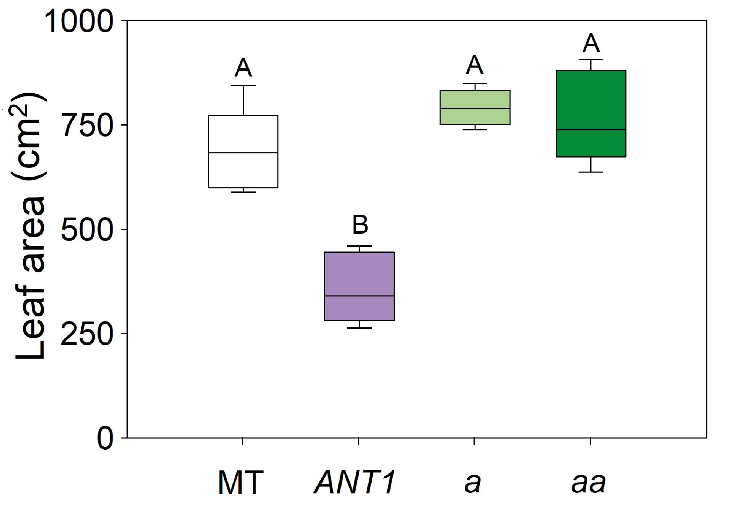


**Figure S1. Increased anthocyanin content reduces leaf area.**  Tomato cv. Micro-Tom (MT); *ANTHOCYANIN* (*ANT1*) anthocyanin accumulator, *anthocyaninless (a)* and *anthocyanin absent* (*aa*). Boxes represent means ± s.e.m. (n=5). Boxes represent interquartile range (IQR), center line the mean, and the ends of the whisker are set at 1.5*IQR above the third quartile and 1.5*IQR below the first quartile. Significant differences tested with one‐way analysis of variance followed by Tukey's honestly significant difference (HSD) test; letters indicate significant differences, *p* <0 .05


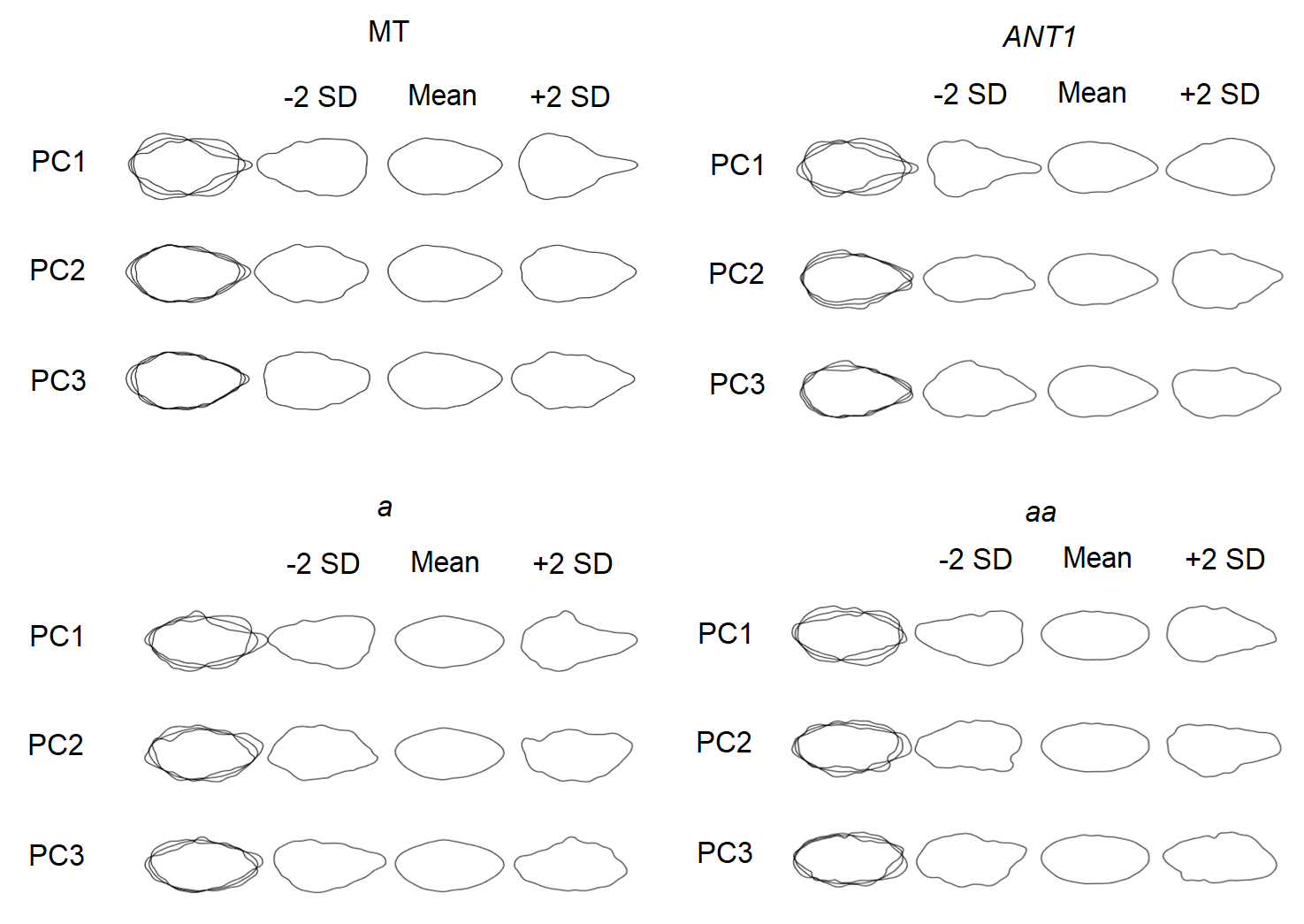


**Figure S2. Anthocyanins impact leaf morphology in tomato**. Principal components display variance in leaflet shape between genotypes. These PC were resulting from 19 leaflets.


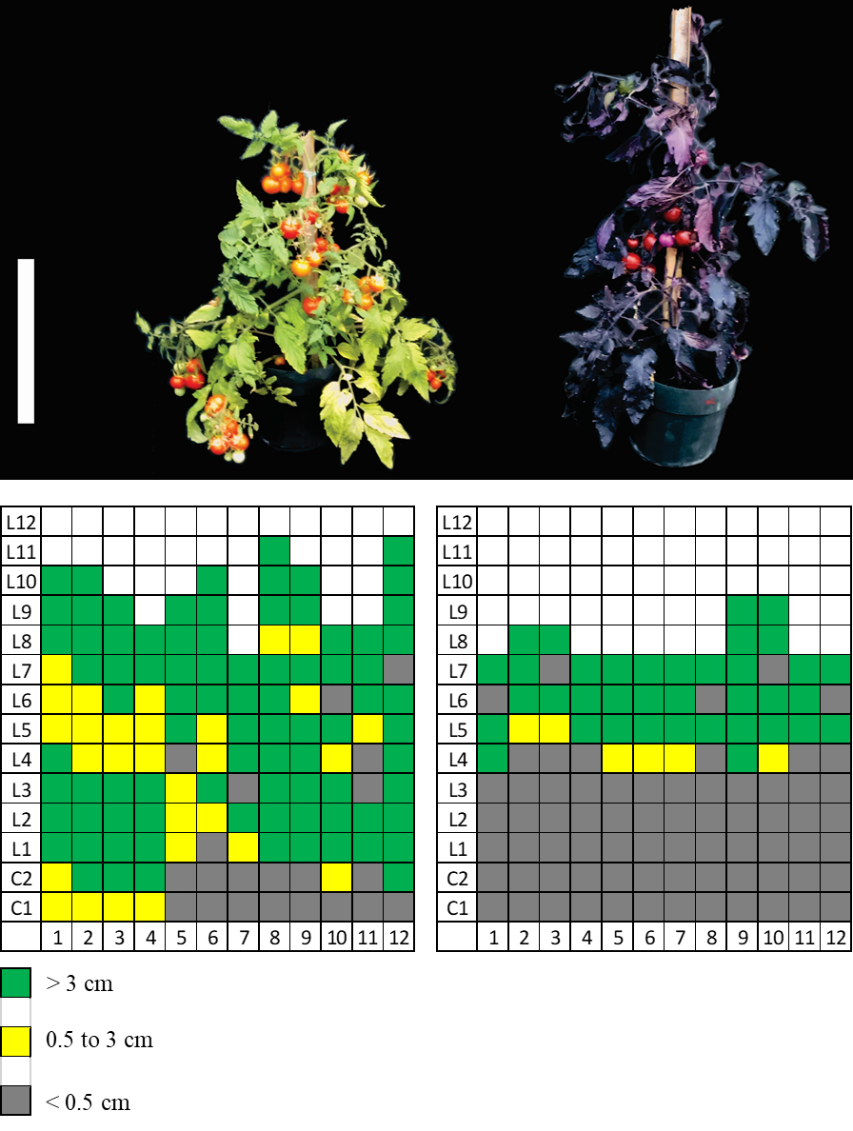


**Figure S3. *ANT1* shows reduced side branching in a hybrid tomato cv. M82 × Micro-Tom (MT) genetic background.** Representative plant phenotypes 45 days after germination. M82 × MT (left); M82 × *ANT1* (right). Scale bar = 30 cm. Branching rate - Each column represents one plant (1-12) and each row an individual bud axil of cotyledon (C1-C2) or leaf (L1-L12). Colors represent the size of the bud as shown in the key.


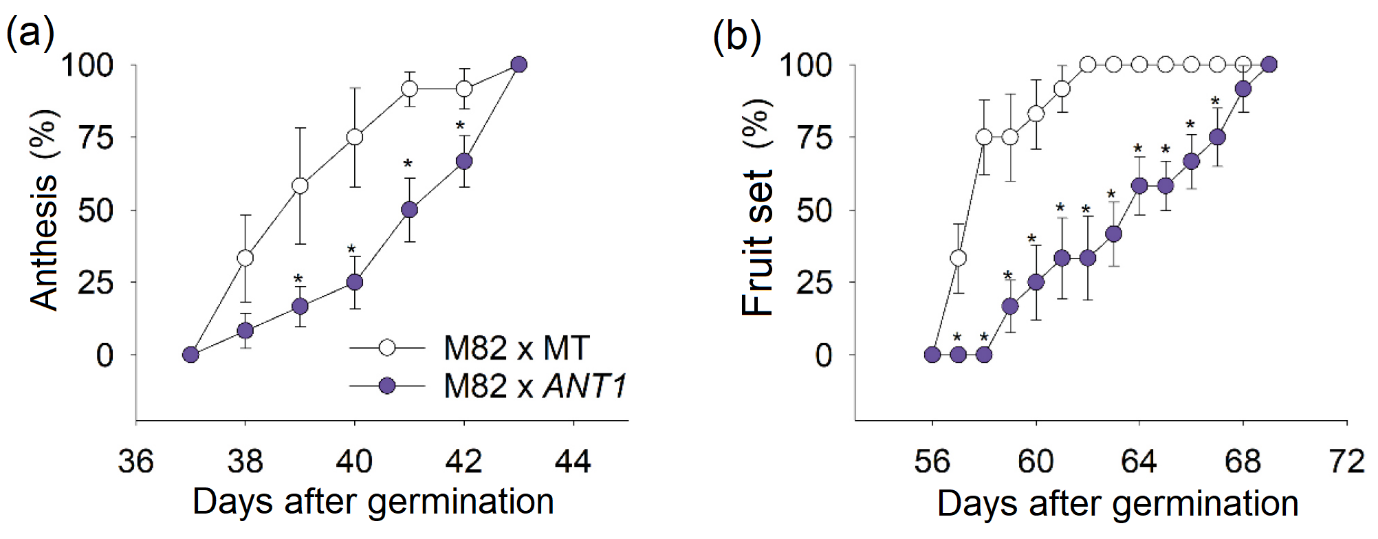


**Figure S4.** **Anthocyanin overproduction impacts flowering and fruit set in a hybrid tomato cv. M82 × Micro-Tom (MT) genetic background. (a)** Rate of anthesis, (b) fruit set rate. Bars are means ± s.e.m. (n=6). Significant differences determined by Student’s *t*-test at *p*<0.001 (**).


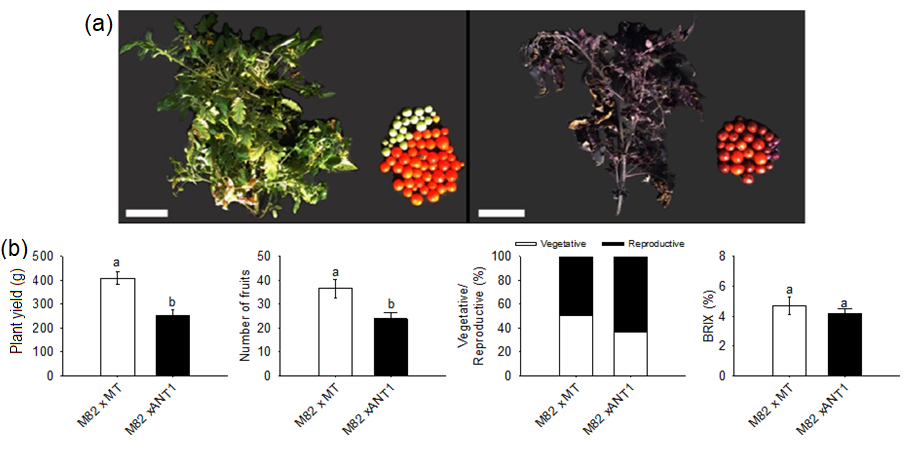


**Figure S5.** **Anthocyanin overproduction impacts productivity in a hybrid tomato background cv M82 × Micro-Tom (MT) (a)** Representative shoots and fruits of 105 days-old hybrid tomato plants (A) M82 × Micro-Tom (left) and M82 × *ANT1^TAL-2^* (right). Bar=10 cm. **(b-e)** Plant productivity parameters. Bars are means ± s.e.m. (n=6). Significant differences determined by Student’s *t*-test at *p*<0.001 (**).


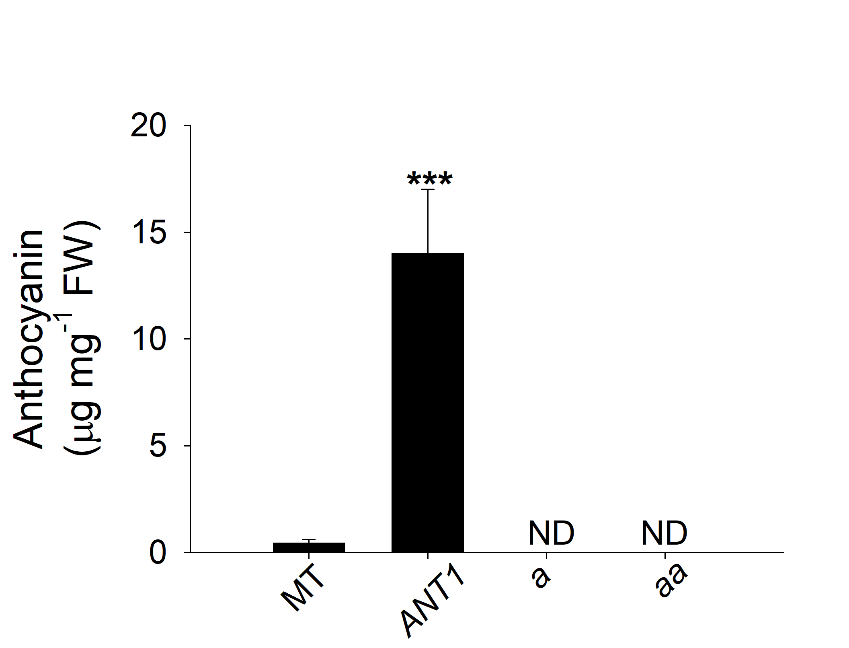


**Figure S6. Increased anthocyanin content in genomically engineered tomato plants.**  Micro-Tom (MT); *ANTHOCYANIN1* (*ANT1*), *anthocyaninless (a)* and *anthocyanin absent* (*aa*). Total anthocyanin quantification in fully expanded leaves. The *a* and *aa* mutants did not reveal any detectable anthocyanin content. Bars are means ± s.e.m. (n=5). ND - not detected. Significant differences tested by *t*-test at *p*<0.001 (***).


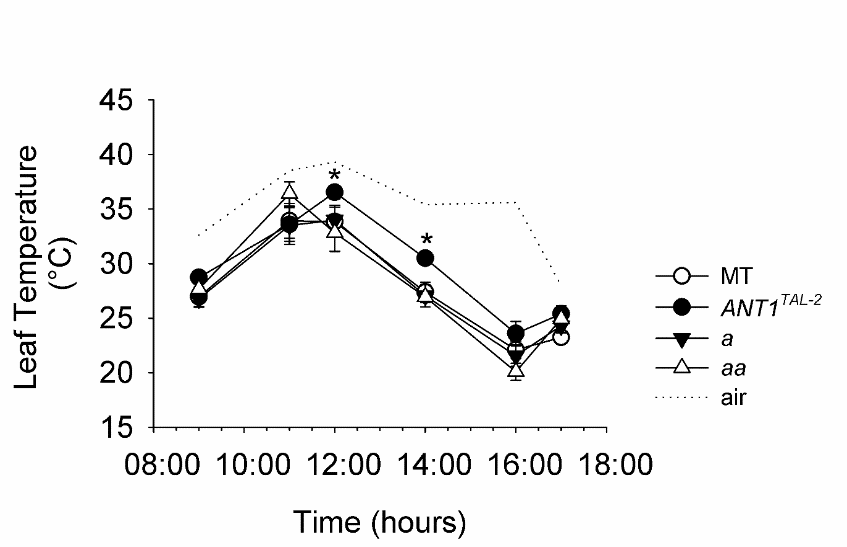


**Figure S7. Leaf temperature is increased in anthocyanin accumulating leaves.** Leaf surface temperature determined throughout the day in Micro-Tom (MT); *ANTHOCYANIN1* (*ANT1*), *anthocyaninless (a)* and *anthocyanin absent* (*aa*). Bars are means ± SE (n=6). Significant differences by *t*-test at *p*<0.05 (*).


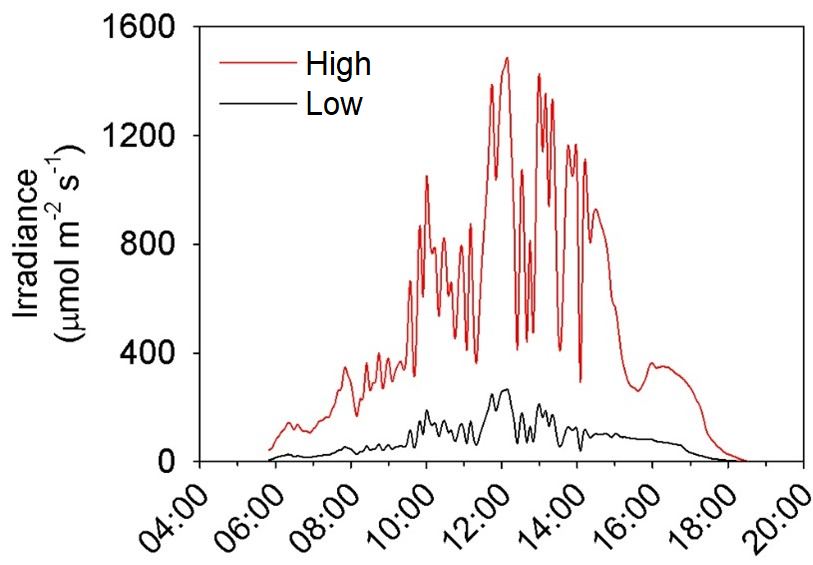


**Figure S8.** **Irradiance throughout a typical day.** The red line represents light incidence on the plants grown in a glasshouse bench (high light treatment), whereas the black line shows the irradiance in plants grown under a neutral shade cloth (low light treatment).
